# Resveratrol decreases CD45^+^CD206^−^ subtype macrophages in LPS‐induced murine acute lung injury by SOCS3 signalling pathway

**DOI:** 10.1111/jcmm.14680

**Published:** 2019-09-27

**Authors:** Lu Hu, Zhihong Chen, Liyang Li, Zhilong Jiang, Lei Zhu

**Affiliations:** ^1^ Department of Pulmonary Medicine Zhongshan Hospital Fudan University Shanghai China

**Keywords:** acute lung injury, macrophages, resveratrol, SOCS3

## Abstract

Acute lung injury/acute respiratory distress syndrome (ALI/ARDS) are life‐threatening condition in critically ill patients. Resveratrol (Res), a natural polyphenol, has therapeutic effect in animal model with ALI; however, whether Res attenuates ALI through modulation of macrophage phenotypes in the animal model remains unknown. We in this study treated LPS‐induced murine ALI with 30 mg/kg Res and observed significantly reduced severity of ALI in the Res‐treated mice 48 hours after Res treatment. Neutrophil infiltrates were significantly reduced, accompanied with lower infiltration of CD45^+^Siglec F^−^ phenotype macrophages, but higher population of CD45^+^Siglec F^+^ and CD45^+^CD206^+^ alternatively activated macrophages (M2 cells) in the Res‐treated mice with ALI. In addition, the expression of IL‐1beta and CXCL15 cytokines was suppressed in the treated mice. However, Res treatment in mice with myeloid cell‐restricted SOCS3 deficiency did not significantly attenuate ALI severity and failed to increase population of both CD45^+^Siglec F^+^ and CD45^+^CD206^+^ M2 subtype macrophages in the murine ALI. Further studies in wild‐type macrophages revealed that Res treatment effectively reduced the expression of IL‐6 and CXCL15, and increased the expression of arginase‐1, SIRT1 and SOCS3. However, macrophages’ lack of SOCS3 expression were resistant to the Res‐induced suppression of IL‐6 and CXCL15 in vitro. Thus, we conclude that Res suppressed CD45^+^Siglec F^−^ and CD45^+^CD206^−^ M1 subtype macrophages through SOCS3 signalling in the LPS‐induced murine ALI.

## 1 INTRODUCTION

Acute respiratory distress syndrome (ARDS) is an acute diffuse pulmonary exudative disorder in critically ill patients. Pulmonary infection and interstitial pneumonia are major causes of the debilitating disease with a high mortality rate of up to 40.7% in China.^1^ Immune system plays a critical role in the progression of ARDS. Neutrophils, macrophages and T lymphocytes are dominant immune cell infiltrates in the inflamed lung tissues, contributing to the progression of ARDS. Macrophages are first‐line cell components in lung tissues, responsible for clearance of invading pathogens and insults. But macrophages/monocytes (MNs) are heterogenous cell types in lung tissues and blood circulation, sharing different cell subtypes. The different macrophage subtypes exclusively release high amount of pro‐inflammatory, anti‐inflammatory cytokines and chemokines, participating in the pathogenesis of ALI/ARDS, asthma and other inflammatory lung diseases.^2^, ^3^

There are no effective therapeutics for ARDS so far. Patients are usually treated with low tidal volume ventilation and fluid restriction,^4^ high dose of corticosteroid^5^ and antioxidants, such as N‐acetylcysteine.^6^ However, the therapeutic approaches produced elusive therapeutic effects and did not improve patient survival rate. Thus, it is essential to explore novel and more effective therapeutics for ARDS.

Resveratrol (Res), a phytoalexin antioxidant from red grapes, has anti‐inflammatory properties.^7^, ^8^ Studies in vivo have confirmed that Res can attenuate the severity of ALI in animal models.^9^, ^10^ Res or Res‐curcumin hybrids can significantly attenuate disease severity of ALI, accompanied with lower production of pro‐inflammatory cytokines and chemokines, such as TNF‐alpha, IL‐1beta, IL‐6, IL‐12, IL‐33, MIP‐2 and IL‐18. In contrast, the anti‐inflammatory cytokines and molecular mediators, such as IL‐10, heme oxygenase‐1 (HO‐1),Nrf2 (nuclear factor‐erythroid 2 related factor) and SOD (superoxide dismutase), were increased in the Res‐treated animal models.^10^, ^11^ The beneficial effects may be associated with up‐regulation and activation of SIRT1, a nicotinamide adenine dinucleotide (NAD+‐dependent deacetylase sirtuin 1).^12^ It was reported that SIRT1 has multiple biological functions on variable cells, by suppressing cell activation, preventing cell senescence, etc. A variable signalling pathways are involved in the beneficial effects, such as NF‐kappa B and JAK/STAT signalling pathway^7^ and PI3K/Nrf2/HO‐1 signalling pathway.^10^ In the Res‐treated cells, the levels of pro‐inflammatory cytokines (TNF‐alpha, IL‐1beta, IL‐6) and chemokines (CCL2/MCP‐1, CCL4/MIP‐1beta, CCL5/RANTES, CXCL10/IP‐10) were significantly suppressed.^13^ However, knockdown of SIRT1 diminished the anti‐inflammatory effects of Res, indicating the critical role of SIRT1 in the Res‐mediated suppression of inflammation.^14^ Furthermore, Res may suppress inflammation through inducing macrophages pyroptosis and apoptosis.^15^, ^16^ Therefore, Res has variable biological function on cells.

IL‐6 and IL‐22 induce activation of signal transducer and activator of transcription‐3 (STAT3) through acetylation in lysine (Lys)685 and phosphorylation in tyrosine (Tyr)705 residues, but activation of STAT3 is suppressed by SIRT1, a class III deacetylase.^17^, ^18^ More activation of STAT3 induces more expression of cytokine expression. Blocking STAT3 activation by small‐molecule STAT3 inhibitor (LLL12) can suppress the expression of IL‐1beta, IL‐6, TNF‐alpha, iNOS, CCL2 and MHC class II in macrophages and inflammatory cells from LPS‐induced ALI mouse model, indicating the pro‐inflammatory function of STAT3.^19^ It is known that SOCS3 is a negative regulator of STAT3. Activation of STAT3 induces transcriptional up‐regulation of SOCS3 and subsequently suppresses STAT3 activation in the inflammatory condition. The negative feedback loop of STAT3/SOCS3 signalling has important implication in overcoming excessive tissue inflammation and preventing tissue damage during inflammatory immune responses.^17^, ^20^ Our previous study and report from other group^21^ indicated that STAT3/SOCS3 signalling suppressed macrophage polarization and activation in the murine ALI mouse model, whereas disruption of STAT3/SOCS3 signalling in SOCS3 myeloid cell‐restricted conditional knock‐out mice (cKO) significantly enhanced the severity of LPS‐induced ALI and sepsis, accompanied with high expression of M1 cell genes (IL‐1beta, IL‐6, IL‐12, IL‐23, inducible NO synthase).^2^, ^20^ However, to our knowledge, whether Res attenuated ALI through macrophage STAT3/SOCS3 signalling remains undefined. In this study, we treated LPS‐induced murine ALI in WT and cKO mice with Res. The results revealed that Res can significantly attenuate the severity and neutrophil infiltration of murine ALI dependent on SOCS3 expression in macrophages. The beneficial results were associated with more CD45^+^CD206^+^ biased M2 macrophage polarization and less infiltration of CD45^+^Siglec F^−^ phenotype macrophages. The results revealed the important role of macrophage STAT3/SOCS3 signalling in the Res‐mediated therapy of ALI.

## 2 MATERIAL AND METHODS

### 2.1 Animal procedure

Eight‐ to ten‐week‐old male wild‐type (WT) C57BL/6 mice were obtained from the Shanghai Biomodel Organism Science & Technology. Animal protocol was reviewed and approved by the laboratory animal care and use committee of Zhongshan Hospital, Fudan University. All animal experiments were performed in the Zhongshan Hospital, Fudan University. SOCS3 cKO were obtained by breeding SOCS3fl/fl mice with Lyz2‐Cre transgenic mice under the control of myeloid cell‐restricted lysozyme 2 (Lyz2) promoter, and the mice with SOCS3^−/−^ phenotype were used for this study.^2^ All mice were treated under anaesthesia with intraperitoneal (ip) administration of 50 mg/kg pentobarbital. Mouse model with ALI was established by intratracheal (i.t.) treatment with 5 mg/kg lipopolysaccharide (LPS) (Sigma‐Aldrich) for 48 hours. To investigate the effects of Res in WT and cKO mice, 30 mg/kg doses of Res in 40% ethanol were ip administered into both WT and cKO mice 3 and 24 hours after LPS i.t. treatment. The mice treated with PBS, LPS or Res alone were used as controls. Lung tissues and bronchoalveolar lavage (BAL) were collected for analysis. The protein in BAL and lung tissue histology were analysed as described previously.^2^

### 2.2 Isolation and culture of bone marrow‐derived macrophages (BMDMs)

To isolate murine BMDMs, bone marrow cells were flushed from the femurs and tibiae of WT or cKO mice. These cells werecultured in RPMI 1640 medium containing 10% FBS and 20 ng/mL of murine M‐CSF (PeproTech) for 7 days to obtain BMDMs. Cell purity was determined by FACS analysis for CD11b and F4/80 (>96%). The cells were pre‐treated with indicated concentration of Res 1 hour prior to LPS 500 ng/mL treatment for 24 hours. The untreated cells and the cells treated Res and LPS alone were used as controls. Gene and protein expression in the treated cells were analysed by qRT‐PCR, Western blot analysis.

### 2.3 Flow cytometry

Lung digests were obtained by incubation with 1 mg/mL collagenase A, and 100 ng/mL DNase (Sigma) for 1 hour. 0.5‐1 × 10(6) cells from lung digests and BAL were stained with cell surface antibodies including PerCP‐cy5.5‐conjugated anti‐F4/80, PE‐Cy7‐conjugated anti‐Ly6G, PE‐conjugated anti‐CD45, BV421‐conjugated anti‐Siglec F and APC‐conjugated anti‐CD206 (BioLegend). Analysis was performed on FACScan cytometer (Becton Dickinson). All data were analysed on FlowJo software (Tree Star).

### 2.4 ELISA assay for cytokines

IL‐1beta and CXCL15 protein concentration in BAL and lung digests were measured by ELISA kit according to the manufacturer's instructions (R&D systems Inc).

### 2.5 Western blot analysis

The concentration of acetylated and phosphorylated STAT3, total STAT3 and SIRT1 protein in lung tissue extracts and macrophage lysates were analysed by Western blot analysis. The procedure was previously described.^2^ Primary antibodies for Western blot analysis include rabbit anti‐total STAT3 (Clone: 79D7), rabbit anti‐phosphorylated STAT3 at residue Tyr‐705, rabbit anti‐acetylated STAT3 at residue Lys‐685, rabbit anti‐SIRT1 (Hangzhou HuaAn Biotech). The anti‐mouse GAPDH antibody was used as a loading control. The blots were washed with TBST buffer and incubated with horseradish peroxidase (HRP)‐conjugated anti‐rabbit immunoglobulin (Ig) and then developed with ECL substrate solution (Amersham Biosciences). Protein expression was quantitatively analysed on ImageJ software by the ratio of densitometric intensity of target protein to internal control GAPDH.

### 2.6 Immunostaining assay

Acetylated STAT3 expression and SIRT1 expression in the lung tissues of treated mice and cells were analysed by immunostaining assay. Briefly, lung tissue sections and cells were fixed with 4% paraformaldehyde, followed by incubation with 0.05% Triton X‐100 and blocking buffer (10% goat serum in PBS). The sections and cells were incubated with first antibody rabbit anti‐acetylated STAT3 and SIRT1 (1:200 dilution) for 3 hours and followed by secondary antibody HRP‐conjugated anti‐rabbit IgG or Cy3‐conjugated anti‐rabbit IgG (1:500 dilution) for 2 hours. The positive cells were developed by 3,3'‐diaminobenzidine (DAB) and visualized under contrast microscope or fluorescence microscope.

### 2.7 Quantitative RT‐PCR (qRT‐PCR)

CXCL15, IL‐1beta IL‐6, SIRT1, arginase‐1, iNOS and SOCS3 mRNA transcripts in lung tissues and cells were analysed by qRT‐PCR as previously described.^2^ The primer sequences were listed in Table 1. The relative gene expression was analysed using the 2^−ΔΔCT^ method.

**Table 1:** Primers used for detection of gene expression in mouse species

Gene	Sense (5′‐3′)	Antisense (5′‐3′)
CXCL15	TGGATCCTGATGCTCCATGG	AGAGGCTTTTCATGCTCAAG
IL‐1beta	AGAGCTTCAGGCAGGCAGTA	AGGTGCTCATGTCCTCATCC
IL‐6	CAACGATGATGCACTTGC	GTACTCCAGGTAGCTATG
SIRT1	TGTTGACCGATGGACTCCTC	ATTGTTCGAGGATCGGTGCC
Arginase‐1	CATTGGCTTGCGAGACGTAGAC	GCTGAAGGTCTCTTCCATCACC
iNOS	ACATCGACCCGTCCACAGTAT	CAGAGGGGTAGGCTTGTCTC
S0CS3	GATTTCGCTTCGGGACTAG	CGGCGGCGGGAAACTTGCTG
GAPDH	TGTTCCTACCCCCAATGTGT	TGTGAGGGAGATGCTCAGTG

### 2.8 Statistical analysis

Results are presented as mean ± standard error (SE) for each group. Student's *t* test was used to determine statistical significance between two groups. One‐way analysis of variance (ANOVA) followed by Tukey's multiple comparisons test was performed for parametric multivariable analysis on GraphPad Prism 7 software. The data were considered statistically significant for *P* values < .05.

## 3 RESULTS

### 3.1 Res attenuated acute lung inflammation in mice with LPS‐induced ALI

**Figure 1:**
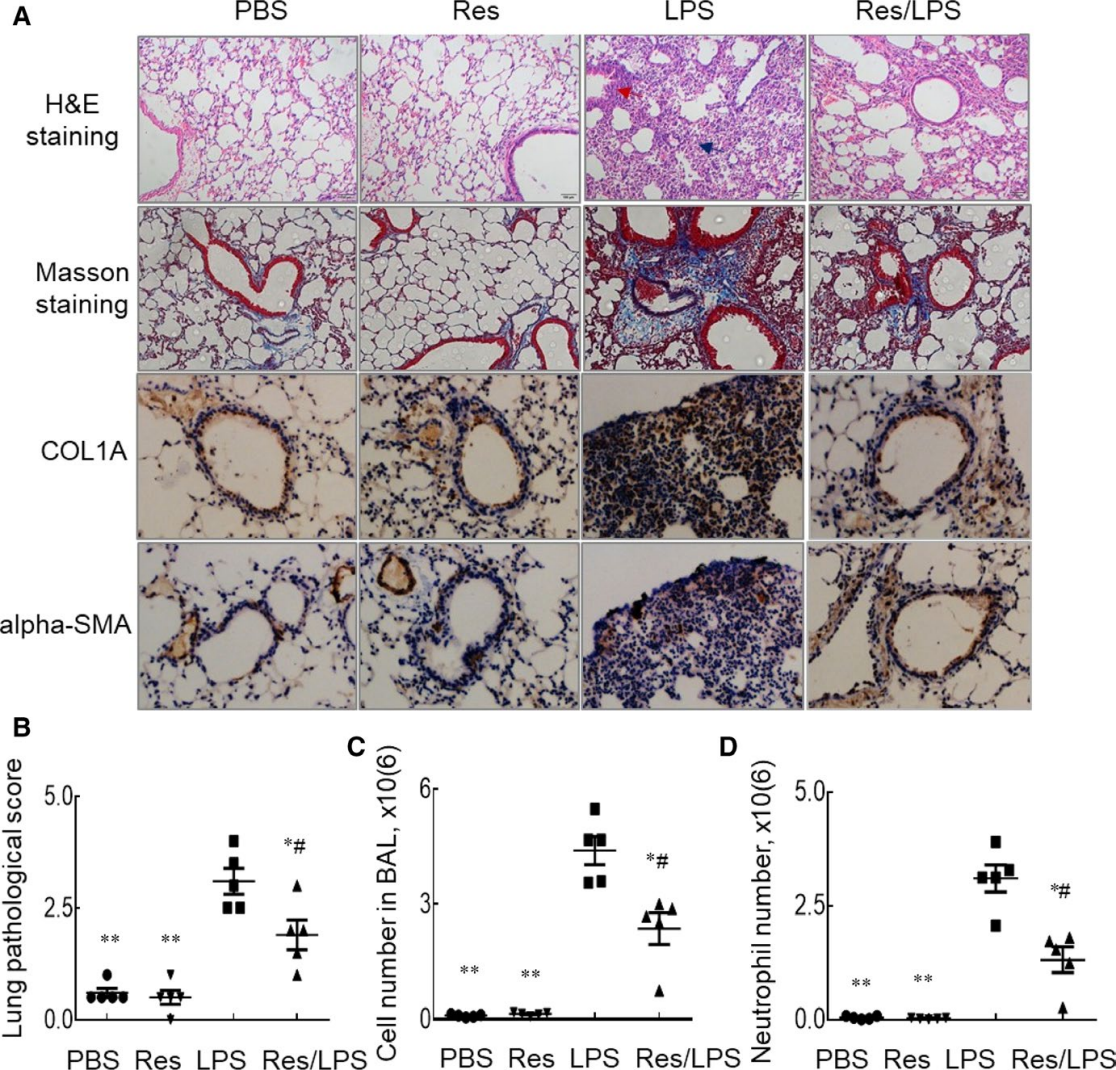
Resveratrol (Res) suppressed acute lung inflammation and fibrosis in wild‐type mice with acute lung injury. Wild‐type (WT) mice with acute lung injury (ALI) were established by i.t. administration of 5 mg/kg LPS (LPS group). In Res/LPS group, mice were treated with LPS and 30 mg/kg Res. The mice treated with PBS and Res alone were negative controls. A, Lung histology and inflammation were analysed by H&E staining, and lung fibrosis was analysed by Masson staining. Blue indicates Masson positive staining. COL1A and alpha‐SMA deposition in the lung tissues was detected by immunostaining; brown indicates positive immunostaining. Blue arrow indicates inflammatory infiltrates; red arrow indicates epithelial cell hyperplasia around bronchi in H&E staining. One representative photograph of each group is shown. B, Quantitative analysis of ALI histology by H&E staining. The score of severity was evaluated by scale from 0 to 4 in terms of infiltrating inflammatory cells and alveoli destruction. C, Total cell counts in BAL. D, Quantitative analysis of neutrophil absolute cell number in BAL

**Figure 2:**
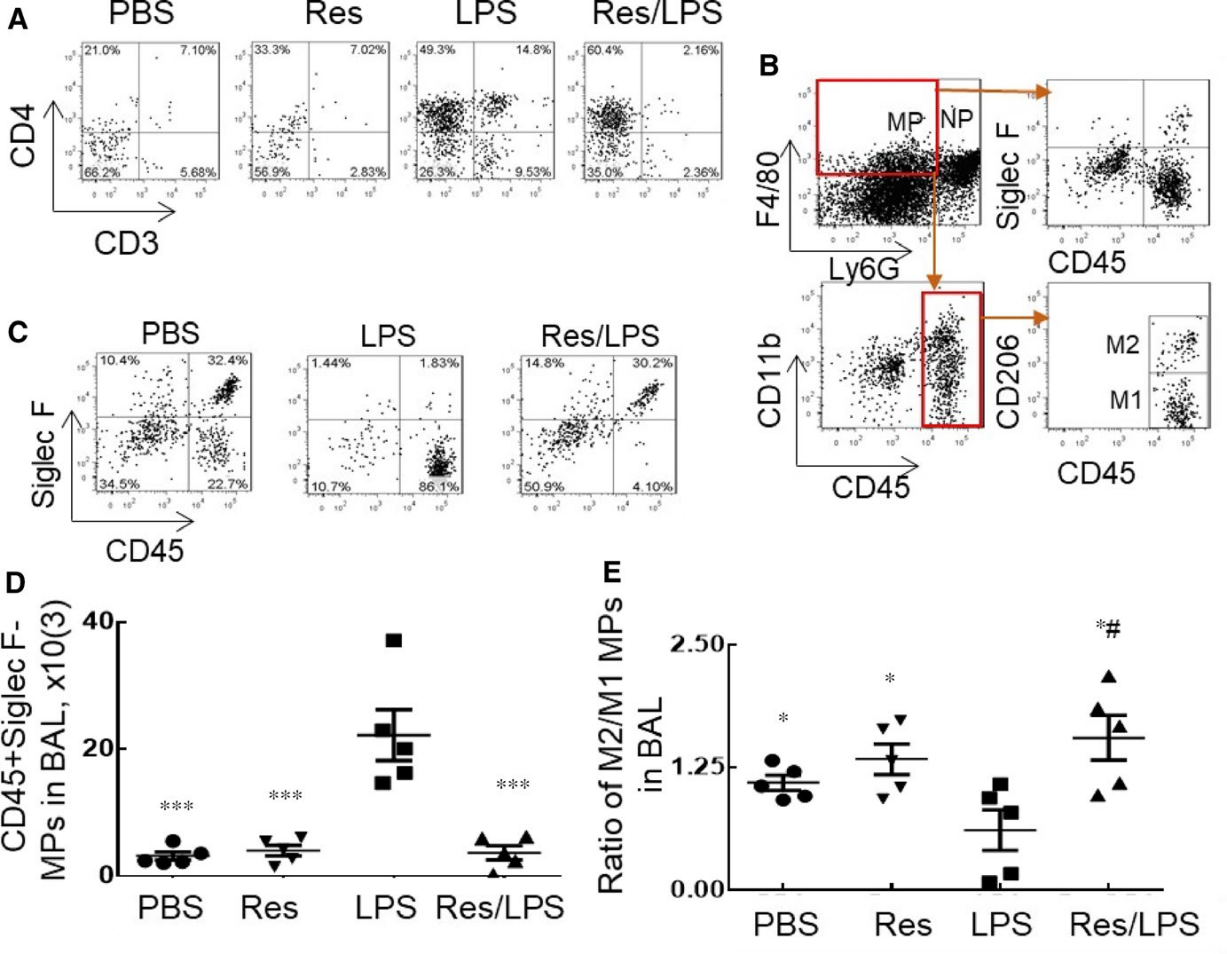
Resveratrol (Res) suppressed infiltration of T lymphocytes and CD45^+^Siglec F^−^ macrophages, but increased M2 subtype macrophages in wild‐type mice with ALI. A, CD3^+^CD4^+^ T lymphocyte influx in BAL were analysed by flow cytometry. One representative dot plot is shown for each group. B, Gating strategy for analysis of neutrophils and macrophage phenotypes by flow cytometry. Neutrophils (NPs) and macrophages (MPs) were identified as F4/80(low)Ly6G^+^ cells and F4/80(high)Ly6G^−^ cells. Macrophage subtypes were further gated on MPs population, as CD45^+^Siglec F^+^, CD45^+^Siglec F^−^ and Siglec F^−^CD45^−^ cell subtypes. M1 and M2 subtype macrophages were identified as CD45^+^CD206^−^ and CD45^+^CD206^+^ cells, respectively. C, LPS largely increased the percentage of CD45^+^Siglec F^−^ subtype, but it was reversed by Res. One representative dot plot of macrophage phenotypes in BAL is shown. D, Quantitative analysis of CD45^+^Siglec F^−^ MPs number in BAL. E, Res reversed LPS‐induced suppression of M2 cell polarization. The CD45^+^CD206^+^ M2 and CD45^+^CD206^−^ M1 subtype MPs in BAL were quantitatively analysed. Data were presented as a ratio of M2/M1 and statistically analysed by one‐way analysis of variance (ANOVA) followed by Tukey's multiple comparisons test. * P < .05, **P < .01 vs LPS group; #* P* < .05 vs PBS group

To investigate the effects of Res on murine ALI and underlying immunological mechanisms, 30 mg/kg Res (Res/LPS group) was i.p. administered into mice 3 and 24 hours after i.t. LPS treatment. The mice treated with PBS, Res and LPS alone were used as naïve and untreated ALI controls. Forty‐eight hours after LPS treatment, we observed the significantly enhanced lung inflammatory infiltrates, epithelial cell hyperplasia and greater lung fibrosis in the LPS‐treated mice. LPS treatment induced more deposition of collagen type 1 alpha A (COL1A) and alpha‐smooth muscle actin (alpha‐SMA) around bronchi and within inflammatory infiltrate loci as analysed by Masson staining and immunostaining. However, co‐treatment with both Res and LPS significantly reversed the severity of ALI, in association with the reduced acute lung inflammation, COL1A and alpha‐SMA deposition (Res/LPS group), compared to the mice treated with LPS alone (Figure 1A,B). Consistent with the results above, the total cell counts (Figure 1C), absolute number of neutrophils (Figure 1D) and percentage of CD3^+^CD4^+^ T cells (Figure 2A) were significantly increased in BAL of mice with ALI, but they were markedly reduced in the mice co‐treated with Res and LPS.

To further investigate the changes of lung macrophages and subtypes associated with ALI suppression by Res, we analysed macrophages and their phenotypes in the treated mice. F4/80(high)Ly6G^−^ cells were identified as lung macrophages (MPs). MPs were further divided into CD45^+^Siglec F^+^, CD45^+^Siglec F^−^ and Siglec F^−^CD45^−^ subtypes by multicolour flow cytometry analysis. In addition, alternatively activated macrophages (M2 cells) and classically activated macrophages (M1 cells) were identified as CD45^+^CD206^+^ and CD45^+^CD206^−^ subtypes (Figure 2B). Further analysis revealed that M2 and M1 cells were equivalent to the population of CD45^+^Siglec F^+^ and CD45^+^Siglec F^−^ subtypes. Study in vivo showed that LPS treatment largely increased the percentage CD45^+^Siglec F^−^ infiltrating macrophages from 22.7% in PBS‐treated control mice to 86.1% in LPS‐treated mice. However, Res treatment largely decreased the percentage of CD45^+^Siglec F^−^ infiltrating macrophages, comparable to the cell percentage in naïve control mice (Figure 2C). The results were confirmed by quantitative analysis, showing the significantly decreased infiltrating CD45^+^Siglec F^−^ macrophage population in the Res/LPS co‐treatment group compared to the LPS‐treated group (Figure 2D, *P* < .01 vs LPS group). Further analysis of M1 and M2 macrophage subtypes showed that CD45^+^CD206^−^ phenotype M1 macrophages were increased, but CD45^+^CD206^+^ phenotype M2 macrophages were decreased in the mice of LPS group. However, the M1‐biased macrophage polarization in LPS‐treated mice was significantly reversed in the mice co‐treated with Res and LPS, showing an increased ratio of M2/M1 cells in this group, comparable to the ratio in PBS and Res alone‐treated group (Figure 2E, *P* < .05). Thereby, Rev attenuated murine ALI, in association with M2‐biased macrophage polarization in the treated lung tissues.

### 3.2 Depletion of M2 macrophages prevented Res‐induced attenuation of ALI

**Figure 3:**
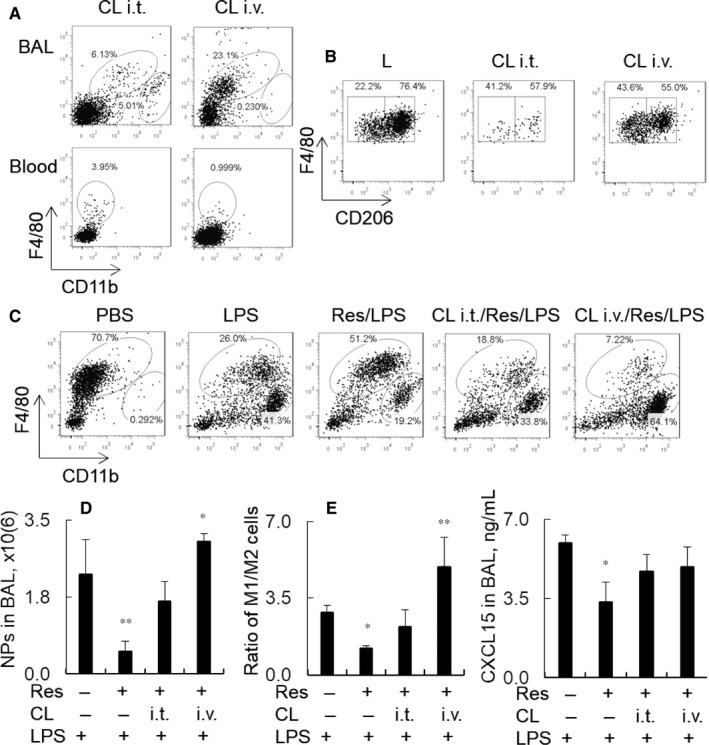
Pre‐depletion of M2 macrophages reversed the suppressive effects of Res on lung inflammation of mice with ALI. A, Naïve mice were i.t. or i.v. administered with 200 μL clodronate liposome (CL) or liposome control (L). Macrophage depletion in BAL and peripheral blood was analysed by flow cytometry analysis. F4/80(high)CD11b(low) and F4/80(low)CD11b(high) were identified as macrophages and NPs. B, M2 and M1 subtype macrophages were analysed in BAL after macrophage depletion. CD206^+^ cells were identified as M2 cells and CD206^−^ cells were identified as M1 cells. C, Mice were pre‐treated with 200 μL CL via i.t. or i.v. for 2 d, and then treated with 5 mg/kg LPS i.t. and 30 mg/kg Res i.p., respectively, for 2 d in CL i.t./Res/LPS and CL i.v./Res/LPS groups. The mice treated with PBS, L i.t./LPS (LPS group) and L i.t./Res/LPS (Res/LPS group) were controls. Representative dot plot in each group is shown. D, Quantitative analysis of F4/80(low)Ly6G(high) NPs in BAL. Data were presented as mean ± standard error, n = 5, *P < .05 vs LPS group. E, M1 and M2 subtype macrophages in lung digests and CXCL15 protein expression in BAL were, respectively, analysed by flow cytometry and ELISA analysis. Data were presented as mean ratio of M1/M2 and expression of CXCL15 ± standard error, n = 5, *P < .05 vs LPS group

To further investigate the involvement of M2 phenotype macrophages in the attenuation of murine ALI by Res, we depleted alveolar macrophages (AMs) and circulating monocytes (MNs) by i.t. and intravenous (i.v.) administration of clodronate liposome (CL), respectively, into the mice with ALI. The mice administered with CL i.t. and CL i.v. alone were used as macrophage depletion control. Two days after CL administration alone, we observed over 90% F4/80(high)CD11b(low) AMs were depleted in BAL and 85% F4/80(high)CD11b(low) MNs were depleted in peripheral blood monocytes (Figure 3A). CL i.t. and CL i.v. both led to 60% reduction of M2/M1 ratio in AMs (Figure 3B), indicating that CL predominantly depleted M2 macrophages in the lungs. Not only CL i.v. predominantly depleted M2 cells of MNs, but also predominantly depleted M2 cells of AMs. Our further study in vivo showed that predominant M2 cell depletion by CL significantly reversed the anti‐inflammatory effects of Res in the mice with ALI, as demonstrated by comparable percentage and absolute number of F4/80(low)CD11b(high) neutrophils (NPs) between LPS and CL i.t./Res/LPS groups. Furthermore, CL i.v. administration induced more neutrophil influx into the lung tissues of CL i.v./Res/LPS group than those of LPS group (Figure 3C‐D, n = 5). Consistent with the results, we observed that predominant depletion of M2 macrophages reversed the suppressive effects of Res on M1 cell polarization and induced higher ratio of M1/M2 cells in CL i.v./Res/LPS groups than LPS and CL i.t./Res/LPS groups (Figure 3E). In addition, there was a trend of more expression of CXCL15 in CL i.v./Res/LPS and CL i.t./Res/LPS groups than those in Res/LPS group. These results provided solid evidence of M2 cell involvement in the Res‐mediated suppression of ALI.

### 3.3 Res attenuated pro‐inflammatory cytokine expression and activation of STAT3

**Figure 4:**
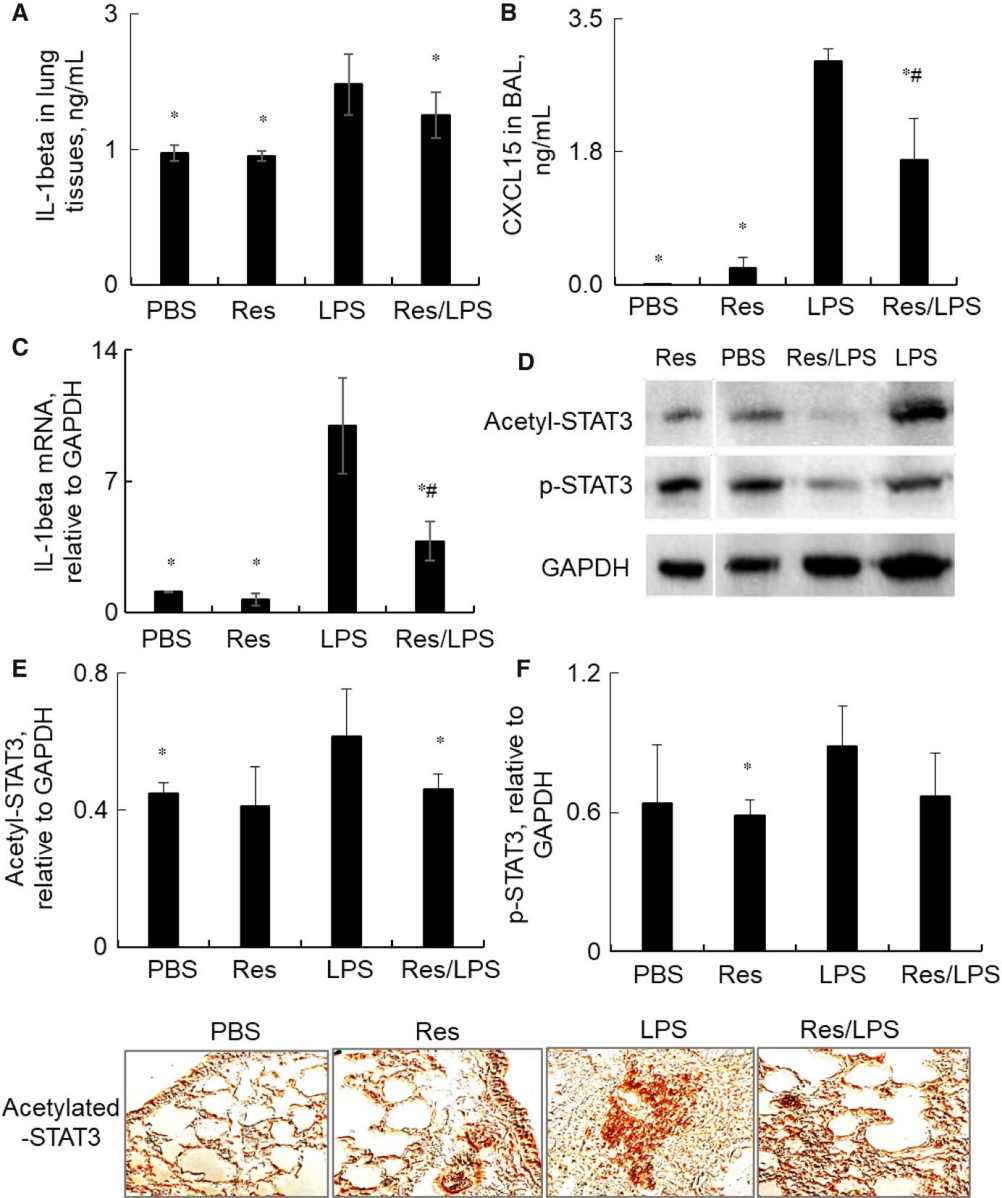
Res attenuated cytokine expression and STAT3 activation in WT mice with ALI. IL‐1beta in lung tissues (A) and CXCL15 (B) protein in BAL were measured by ELISA assay. IL‐1beta mRNA transcripts in lung tissues (C) was quantitatively analysed by qRT‐PCR. Data were presented as mean ΔΔCt relative to GAPDH internal control. n = 6. * *P* < .05 vs LPS group. D, Acetylated STAT3 at Lys‐685, phosphorylated STAT3 at Tyr‐705 and SIRT1 protein expression in the lung tissues were analysed by Western blot analysis. GAPDH was used as loading control. One representative blot is shown. The acetylated (E) and phosphorylated STAT3 (F) were quantitatively analysed by Image J software. Data were presented as ratio of target protein densitometric density to GAPDH. n = 6, * *P* < .05 vs LPS group; #*P* < .05 vs PBS group. Data were statistically analysed by one‐way analysis of variance (ANOVA) followed by Tukey's multiple comparisons test. G, Immunostaining for acetylated‐STAT3 expression in lung tissues. Positive cells were developed by DAB and visualized under contrast microscope. One of representative photograph in each group is shown

CXCL15 attracts neutrophils into the inflamed sites, favouring pro‐inflammatory M1 macrophage polarization.^22^ To investigate whether these mediators are modulated by Res, we measured IL‐1beta and CXCL5 expression in the lung tissues by ELISA (Figure 4A‐B) and qRT‐PCR analysis (Figure 4C). The results revealed that Res significantly decreased LPS‐induced up‐regulation of IL‐1beta and CXCL5 expression in the lung tissues of mice with ALI (n = 6, *P < .05 vs LPS group). Consistently, more STAT3 was acetylated and phosphorylated at Lys‐685 and Tyr‐705 residues by LPS, but that was moderately attenuated in the lung tissues of mice co‐treated with both Res and LPS, than the mice treated with LPS alone (Figure 4D). Quantitative analysis showed that acetylation of STAT3 was significantly decreased (*P* < .05) and there was a trend of reduced phosphorylation of STAT3 in the Res/LPS group compared to the mice treated with LPS alone (Figure 4E‐F). The results were consistent with the results of lung tissue immunostaining, in which the acetylated STAT3^+^ infiltrating inflammatory cells were increased around bronchi in the lung tissue section of mice treated with LPS alone, but few of the acetylated STAT3^+^ cells was observed in the Res/LPS‐treated mice (Figure 4G). Thus, Res treatment protected mice from LPS‐induced ALI possibly through suppressing STAT3 acetylation and inducing M2‐biased polarization of subtype macrophages.

### 3.4 Res attenuated ALI by STAT3/SOCS3 signalling pathway

**Figure 5:**
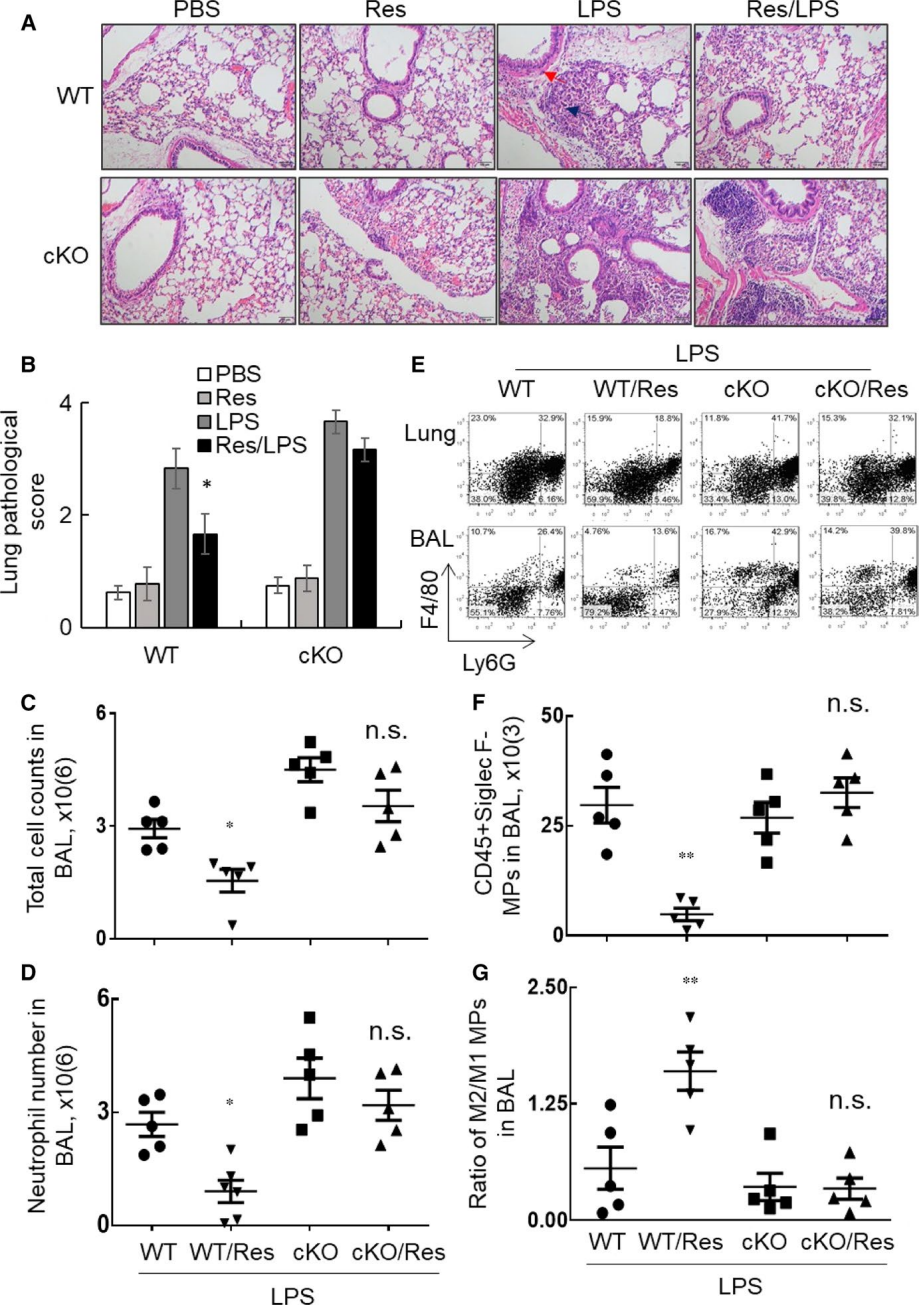
Res did not attenuate ALI in SOCS3 conditional knock‐out mice. Wild‐type (WT) and SOCS3 conditional knock‐out (cKO) mice were i.t. treated with 5 mg/kg LPS on day 0. 30 mg/kg Res were i.p. administered at 3 and 24 h after LPS treatment. The mice treated with PBS, Res and LPS alone were used as controls. A, Representative photograph of lung tissue histology by H&E staining. Acute inflammatory infiltration, alveoli destruction (Blue arrow), epithelial cell hyperplasia (Red arrow) were attenuated in the Res/LPS group of WT mice, but not in cKO mice compared to LPS control. B, Quantitative analysis of lung pathology by scale from 0 to 4 in terms of infiltrating inflammatory cells and alveoli destruction. C, Total cell counts and (D) neutrophil absolute cell number in BAL were calculated. E, F4/80(high)Ly6G^−^ macrophages and F4/80(low)Ly6G^+^ neutrophils in lung tissues and BAL were analysed by flow cytometry analysis. One of representative dot plot is shown. F, Absolute number of CD45^+^Siglec F^−^ MPs in BAL. G, CD45^+^CD206^+^ M2 and CD45^+^CD206^−^ M1 subtype MPs in BAL were quantitatively analysed and data were presented as a ratio of M2/M1. Data were statistically analysed by one‐way analysis of variance (ANOVA) followed by Tukey's multiple comparisons test. * *P* < .05, **P < .01 vs the Res‐untreated WT or cKO groups. n.s. was not statistically significant

Our previous study indicated that STAT3/SOCS3 signalling pathway played an important regulatory role in the progression of ALI.^3^ To investigate whether Res suppresses ALI through STAT3/SOCS3 signalling, we compared the therapeutic effects of Res in WT and cKO mice, in which SOCS3 was myeloid cell‐restricted depleted. The mice untreated, Res or LPS treated alone were used as naïve or untreated controls. The results revealed that LPS i.t. induced more lung inflammation and injury in cKO mice than those of WT mice as previously reported.^3^ Though Res can effectively suppress the severity of ALI and lung inflammation in WT mice, myeloid cell‐restricted lack of SOCS3 prevented the suppressive effects of Res on cKO mice with ALI (Figure 5A‐B). The results were consistent with the results of total cell counts (Figure 5C) and absolute number of neutrophils (Figure 5D) in BAL. Flow cytometry analysis showed that the percentage of F4/80(low)Ly6G^+^ phenotype neutrophils in BAL and lung tissues in WT mice with ALI were significantly suppressed, but not in those of cKO mice (Figure 5E). More studies on macrophage subtypes by flow cytometry showed that the population of CD45^+^Siglec F^−^ subtype infiltrating macrophages was significantly reduced in the lung of Res‐treated WT mice with ALI (*P* < .05), but not in the lung of cKO mice with ALI (Figure 5F). Furthermore, we observed the heightened ratio of M2/M1 subtype macrophages in the Res‐treated WT mice, but not altered in the Res‐treated cKO mice (Figure 5G). Therefore, myeloid cell‐restricted SOCS3 deficiency prevented Res‐mediated suppression of ALI through STAT3/SOCS3 signalling pathway.

**Figure 6:**
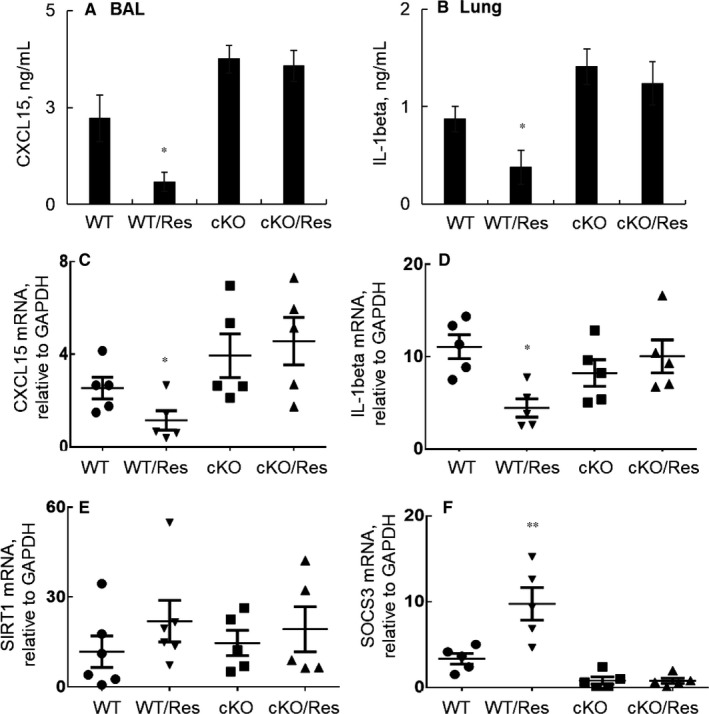
Res did not attenuate the expression of inflammatory cytokines and chemokine in SOCS3 cKO mice with LPS‐induced ALI. A,Protein concentration of CXCL15 (A) in BAL and IL‐1beta (B) in lung tissues were analysed by ELISA. Data were presented as mean ± standard error among each group. C, CXCL15; D, IL‐1beta; E, SIRT1 and F, SOCS3 mRNA transcripts in the lung tissues were analysed by qRT‐PCR analysis. Data were presented as mean ΔΔCt relative to GAPDH internal control. n = 5, * *P* < .05, **P < .01 vs the Res‐untreated WT or cKO groups. Data were statistically analysed by one‐way analysis of variance (ANOVA) followed by Tukey's multiple comparisons test

To further investigate whether pro‐inflammatory cytokines and other mediators were affected by Res treatment in cKO mice, we measured CXCL15 and IL‐1beta expression by ELISA assay. The results showed a significantly reduced expression of CXCL15 and IL‐1beta expression at protein (Figure 6A‐B) and mRNA levels (Figure 6C‐D) in LPS‐induced WT mice, but not in LPS‐induced cKO mice (*P < .05 vs Res‐untreated WT or cKO groups). In addition, we observed a trend of increased Sirt1 expression (Figure 6E) and significantly increased SOCS3 expression (Figure 6F) in the LPS‐induced WT mice, but not in LPS‐induced cKO mice. However, SOCS3 expression was not well detected by qRT‐PCR in the cKO lung tissues, confirming the deficiency of SCOCS3 expression in cKO mice. The results indicated that Res suppressed ALI through suggested STAT3/SOCS3 signalling pathway. SIRT1 expression and activation should be involved in the beneficial effects of Res in vivo.

### 3.5 Res suppressed M1 cell‐biased polarization of BMDMs from WT mice, but not from cKO mice

**Figure 7:**
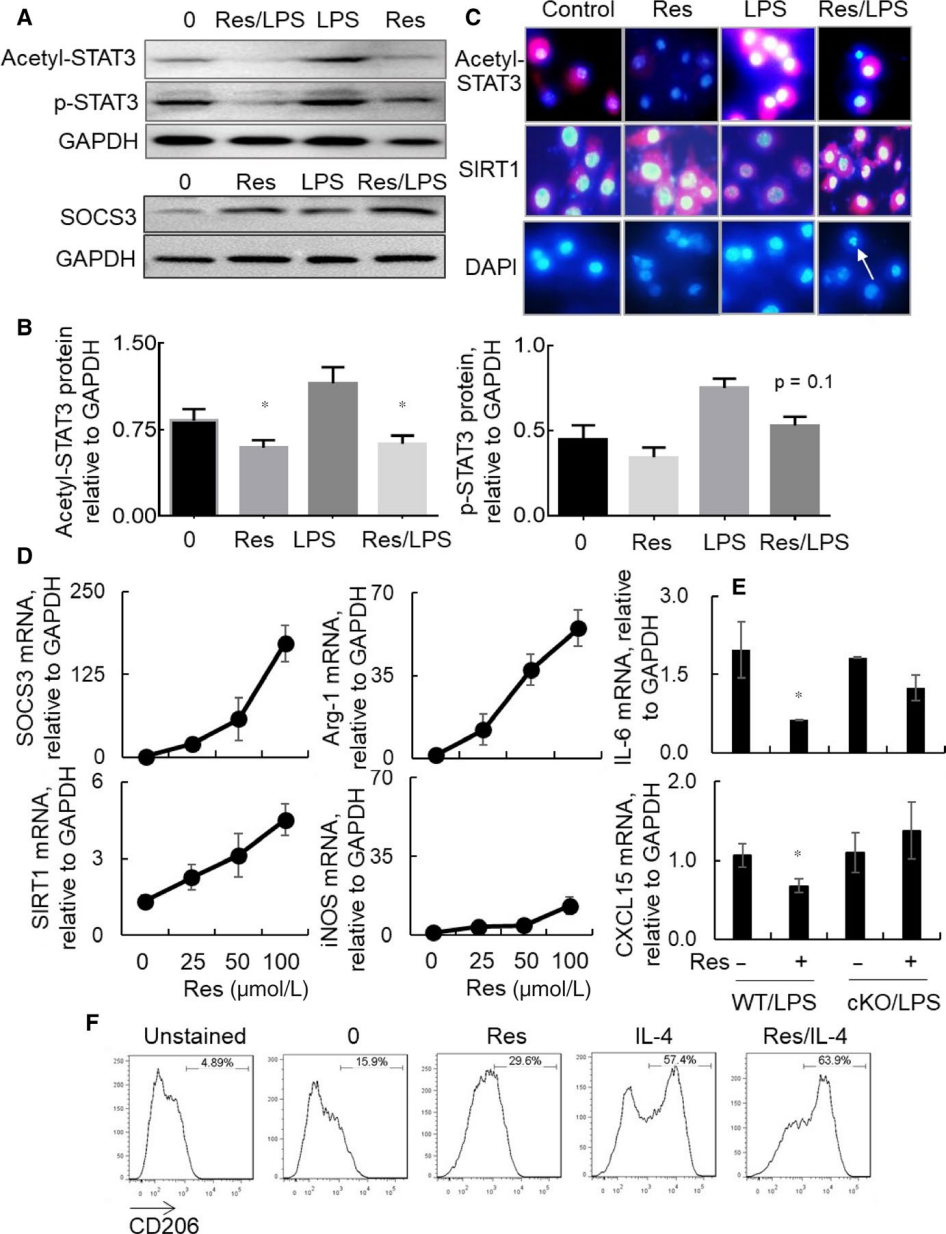
Res suppressed activation and M1 cell‐biased polarization of bone marrow‐derived macrophage (BMDMs) from WT mice, but not from cKO mice. A, Western blot analysis for phosphorylated, acetylated STAT3 and SOCS3 expression in BMDMs 24 h after treatment with 50 µmol/L (upper panel) or 25 µmol/L (lower panel) Res alone or in combination with 500 ng/mL LPS. GAPDH was used as internal loading control. One representative blot is shown. B, Quantitative analysis of phosphorylated‐STAT3 and acetylated STAT3 expression on Western blots by ImageJ software. Data were presented as ratio of target protein densitometric density to loading control GAPDH. *P < .05 vs the Res‐untreated LPS control. Data were statistically analysed by one‐way analysis of variance (ANOVA) followed by Tukey's multiple comparisons test. C, Immunostaining for acetylated STAT3 and SIRT1 expression in the treated BMDMs. The cells were incubated with rabbit anti‐acetylated STAT3 antibody or anti‐SIRT1 antibody and followed by Cy3‐conjugated anti‐rabbit IgG (Red). Nuclei were stained with DAPI (Blue). 50 μmol/L Res suppressed acetylation of STAT3 and increased expression of SIRT1, but induced nuclei damage and cell apoptosis (white arrow). One representative photograph is shown. D, qRT‐PCR analysis for SOCS3, SIRT1, arginase‐1 (Arg‐1) and iNOS mRNA transcripts. The cells were treated with the indicated concentration of Res for 24 h in triplicate wells. Data were presented as mean of ΔΔCt relative to GAPDH ± standard error. E, qRT‐PCR analysis for IL‐6 and CXCL15 mRNA transcripts in WT or SOCS3 cKO mice‐derived BMDMs 24 h after treatment with 500 ng/mL LPS with or without 50 µmol/L Res pre‐treatment. Data were presented as mean ΔΔCt relative to GAPDH ± standard error, n = 3. *P < .05 vs Res‐untreated control. F, Flow cytometry analysis for CD206 expression on BMDMs 24 h after treatment with 50 µmol/L Res and 20 ng/mL IL‐4 alone or both. The cells stained with anti‐F4/80 and anti‐CD11b antibodies without addition of anti‐CD206 antibody were unstained controls. CD206^+^ cells were gated on F4/80^+^CD11b^+^ macrophages. One representative histogram is shown for each treatment

To further confirm whether the suppressive effects of Res in murine ALI are mediated by STAT3/SOCS3 signalling in macrophages, we treated BMDMs with different concentrations of Res. The results indicated that LPS activated STAT3, inducing phosphorylation and acetylation of STAT3, but the STAT3 activation was moderately suppressed by Res treatment in BMDMs (Figure 7A‐C). Because SOCS3 is a negative feedback regulator of STAT3, our further analysis by Western blot and qRT‐PCR analysis revealed that Res largely up‐regulated expression of SOCS3 in BMDMs, that was negatively correlated to the suppression of STAT3 (Figure 7A,D). In addition, we observed lower expression of IL‐6 and CXCL15 (Figure 7E), but higher expression of SIRT1 and arginase‐1 (Figure 7C‐D) in the Res‐treated BMDMs. Though both iNOS1 and arginase‐1 were increased after Res treatment, Res induced fivefold more increases in arginase‐1 than iNOS1, indicating a M2‐biased macrophage polarization by Res. To support the results, our further analysis by flow cytometry analysis showed that Res induced twofold more expression of CD206, a M2 cell‐specific marker in the Res‐treated BMDMs than the untreated cells. IL‐4 is a potent M2 cell inducer. Res and IL‐4 additively increased the CD206 expression in BMDMs (Figure 7F). The results indicated that Res suppressed activation of macrophages and improved M2‐cell‐biased polarization. However, the suppressive effects of Res on macrophage activation and M1 cell‐biased polarization were significantly abolished in BMDMs‐derived from SOCS3 conditional knock‐out mice, because Res failed to inhibit the expression of IL‐6 and CXCL15, their expression levels were comparable between BMDMs‐derived from WT and SOCS3 cKO mice after Res treatment (Figure 7E). Therefore, SOCS3 signalling was critically involved in Res‐mediated suppression of macrophage polarization in vitro. Res increased M2‐biased BMDM polarization and suppressed pro‐inflammatory cytokine expression possibly through STAT3/SOCS3 signalling pathway.

In addition to the suppressive role of Res in macrophage activation, we also observed the increased cell apoptosis in the Res‐treated macrophages. The apoptotic cells were presented as degraded nuclei and cell necrosis as shown by DAPI nuclei staining (Figure 7C, white arrow). The increased macrophage apoptosis by Res was associated with the reduced cell proliferation and cell survival in vivo. Our additional cell adoptive transfer study showed low population of PKH26‐labelled exogeneous Res‐treated WT BMDMs than the untreated exogenous WT BMDMs in the lung tissues of mice with LPS‐induced ALI (data not shown). Thereby, Res suppressed ALI possibly through inducing macrophage apoptosis that explained lower infiltrating CD45^+^Siglec F^−^ and M1 subtype macrophages in the lung of Res‐treated mice.

## 4 DISCUSSION

Macrophages are heterogeneous cell components and play an important role in the pathogenesis of ALI/ARDS. There are different macrophage subtypes with distinct biological function. Modulation of macrophage subtypes is a promising therapeutic approach in the treatment of many inflammatory diseases such as ALI/ARDS. In the LPS‐induced murine ALI model, Res can effectively attenuate the severity of ALI, and the beneficial effects are associated with lower NLRP3 inflammasome activation and release of IL‐1beta and IL‐18.^11^ However, whether Res suppresses ALI through modulation of macrophage subtypes and whether STAT3/SOCS3 signalling is involved in the therapeutic effects remains unknown.

In this study, we investigated the role and molecular mechanisms of Res in the therapy of ALI. Our results showed that Res significantly attenuated LPS‐induced ALI and inflammatory responses in WT mice. The therapeutic effects were associated with the suppressed STAT3 acetylation in lysine (Lys)685 and phosphorylation in tyrosine (Tyr)705, indicating the involvement of STAT3 activation in the Res therapeutic effects. Meanwhile, SIRT1 and SOCS3 were up‐regulated by Res treatment; whereas IL‐1beta and CXCL15 expression were suppressed by Res in vivo and in vitro. Thus, Res attenuated ALI possibly through activation of SIRT1, subsequently reducing activation of STAT3, consistent with the previous report, in which SIRT1 suppressed Lys 685 acetylation of STAT3 in keratinocytes.^18^

Because CXCL15 is a lung neutrophil attractant, the attenuated levels of CXCL15 should be responsible for lower neutrophil infiltrates in the inflamed lung tissues of the Res‐treated mice. Indeed, our further analysis by flow cytometry showed lower population of neutrophils, reduced infiltrating CD45^+^Siglec F^−^ subtype macrophages, but higher ratio of CD45^+^CD206^+^ M2 cells to CD45^+^CD206^−^ phenotype M1 cells in the lung of Res‐treated mice. We conclude that Res may exclusively target macrophages, particularly M2 cells, because our additional study in vivo showed that Res failed to suppress ALI in the mice with pre‐depletion of M2 subtype macrophages including AMs and MNs. Furthermore, lung inflammation and neutrophil influx were even enhanced in the mice with ALI after pre‐depletion of MNs by CL i.v. treatment, that maybe caused by depletion of M2 cells of both AMs and MNs. In addition, M2‐biased macrophage polarization by Res was further supported by our in vitro study, in which Res induced the expression of M1‐specific maker iNOS and M2‐specific marker arginase‐1 in the treated macrophages. However, Arg‐1 was more induced than did iNOS by Res, indicating a M2‐biased polarization in the Res‐treated cells. Thereby, Res suppressed murine ALI possibly by promoting M2‐biased macrophage polarization, subsequently suppressing release of debilitating pro‐inflammatory cytokines and chemokines.

It is documented that SIRT1 is critically involved in variable cell biological function.^22^, ^23^, ^24^ Loss of SIRT1 in SIRT1 knock‐out mice delayed locomotor recovery, in association with more expression of pro‐inflammatory cytokines and population of M1 type macrophages in a mouse model with spinal cord injury.^25^ Consistent with the previous report,^22^ we observed that Res up‐regulated the expression of SIRT1, whereas LPS moderately reduced the expression of SIRT1 in BMDMs. The results were consistent with previously reports, in which there was lower SIRT1 expression in the inflammatory lungs of animal models with LPS‐induced ALI and pulmonary contusion.^26^, ^27^, ^28^ However, our further study indicated that co‐treatment of BMDMs with both Res and LPS prevented the LPS‐induced suppression of SIRT1 (Figure 7C), demonstrating the important role of Res in activation of SIRT1 signalling.

STAT3/SOCS3 signalling is anti‐inflammatory feedback loop and critically involved in the mouse model with ALI,^2^ important in the control of excessive tissue inflammation and damage. It was previously reported that SOCS3 expression in mononuclear cells was reduced by nutritional supplement containing resveratrol and muscadine grape polyphenols in another animal model.^29^ In medulloblastoma cells, STAT3/SOCS3 signalling was suppressed by Res treatment.^30^ Therefore, Res may exert anti‐inflammatory function through down‐regulating STAT3/SOCS3 signalling. However, it was not well understood whether STAT3/SOCS3 signalling participates in the Res‐mediated therapy of ALI. To address this issue, we in this study treated WT and cKO mice with Res. As expected, we observed a significantly reduced ALI severity in the Res‐treated WT mice, but the beneficial effects were not well observed by treatment with the same doses of Res in cKO mice. The results were accompanied with the suppressed expression of CXCL15 and IL‐1beta in the Res‐treated WT mice, but not in cKO mice, further confirming the protective role of Res in ALI and importance of STAT3/SOCS3 signalling in Res‐mediated therapy of ALI. Because SIRT1 is an important Res downstream regulator, our additional study in vitro showed that SIRT1 and SOCS3 expression were significantly increased by Res treatment, but the expressions of STAT3, IL‐6 and CXCL15 were significantly reduced by Res in WT macrophages. We speculate that SIRT1 is involved in the Res‐mediated regulation of STAT3/SOCS3 signalling in vivo and in vitro. However, the reduced IL‐6 expression and CXCL15 expression in WT macrophages were not observed in the SOCS3‐deficient macrophages, indicating the pivotal role of SOCS3 in the Res‐induced suppression of these pro‐inflammatory cytokines. Res may increase the expression of SOCS3 through up‐regulation of SIRT1. The increased SOCS3 in turn suppresses STAT3 expression and activation, ultimately inhibiting uncontrolled immune responses in the lung of ALI/ARDS.

As previously reported,^16^, ^31^ we also observed that Res induced apoptosis of BMDMs and RAW 264.7 macrophages (data not shown) as evidenced by more Annexin V+ cells at high concentration of Res (>50 μmol/L). We observed more nuclei damage and accumulation of intracellular LC3 protein, a key cell autophagy molecule in the Res‐treated cells (data not shown). Further investigation should be required to dissect the role and underlying molecular mechanisms of Res in macrophage apoptosis in the future.

**Figure 8:**
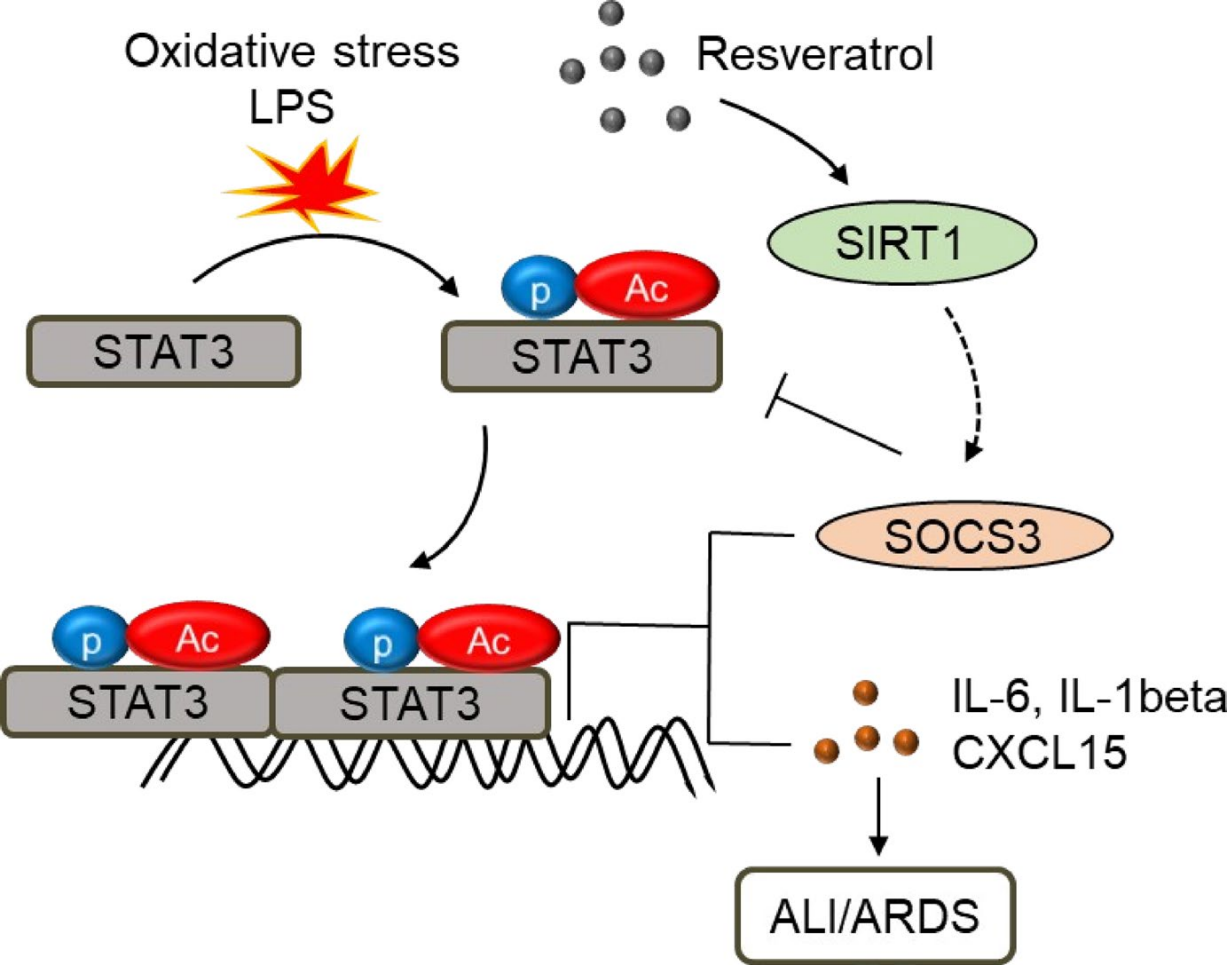
Schematic diagram of resveratrol (Res)‐mediated suppression of ALI through STAT3/SOCS3 signalling. LPS induces oxidative stress and activates STAT3, subsequently induces IL‐6, IL‐1beta and CXCL15 expression, ultimately causes ALI/ARDS. Activation of STAT3 up‐regulates expression of SOCS3 that negatively suppresses STAT3 signalling‐mediated pro‐inflammatory cytokine expression and maintains a balance between pro‐inflammatory and anti‐inflammatory responses. Res increases the expression and activation of anti‐inflammatory SIRT1 and SOCS3, subsequently lead to lower expression of IL‐6, IL‐1beta and CXCL15

In conclusion, this study indicated that Res suppressed lung inflammation of mice with LPS‐induced ALI, in association with significantly reduced neutrophil infiltration and production of pro‐inflammatory cytokines. Res significantly modulated macrophage activation and polarization, with enhanced polarization of anti‐inflammatory M2 and CD45^+^Siglec F^+^ subtype macrophages by Res treatment. STAT3/SOCS3 signalling critically participated in macrophage polarization and mediated the suppressive effects of Res in mice with ALI (Figure 8). Thereby, this study gained insights into a new role and underlying molecular mechanisms of Res in the therapy of murine ALI. The results provided a solid molecular basis for application of Res to clinical trial in the future.

## 5 CLINICAL PERSPECTIVES

- Res has anti‐inflammatory and antioxidant effects. Previous studies showed that Res attenuated the severity of ALI in animal models, associated with lower expression of pro‐inflammatory cytokines. However, whether SOCS3 signalling pathway in macrophages is involved in the attenuation of ALI remains unknown.
- In this study, we compared the effects of Res on lung inflammation of murine ALI and macrophage phenotypes in WT and SOCS3 cKO mice. The results revealed that Res suppressed lung inflammation in murine ALI, associated with the suppressed CD45^+^Siglec F^−^ and CD45^+^CD206^−^ M1 subtype macrophages, but increased CD45^+^Siglec F^+^ and CD45^+^CD206^+^ M2 subtype macrophages in murine ALI. However, the effects were not observed in SOCS3 cKO mice.
- The results gained insights into the important role of STAT3/SOCS3 signalling in polarization of CD45^+^Siglec F^−^ subtype and CD45^+^CD206^−^ M1 subtype macrophages, as well as resveratrol‐mediated attenuation of lung inflammation in murine ALI. Therefore, modulation of macrophage subtypes by targeting SOCS3 signalling is a promising therapeutic approach in the treatment of murine ALI and patients with ARDS in the future.

## CONFLICT OF INTEREST

The authors confirm that there are no conflicts of interest.

## AUTHOR CONTRIBUTIONS

L. Hu, Z. Chen and L. Li participated in Western blot analysis. Z. Jiang participated in the generation of hypothesis, animal experiments, cell culture, data assembly, analysis, manuscript writing and revision, was responsible for overall direction of the work. L. Zhu participated in the generation of hypothesis and data interpretation. All authors read and approved the final manuscript.
